# An evaluation of the hepatitis C testing, care and treatment program in the country of Georgia’s corrections system, December 2013 – April 2015

**DOI:** 10.1186/s12889-019-6783-4

**Published:** 2019-05-10

**Authors:** Aaron M. Harris, Otar Chokoshvili, Joshua Biddle, Kostantine Turashvili, Maia Japaridze, Irma Burjanadze, Tengiz Tsertsvadze, Lali Sharvadze, Marine Karchava, Archil Talakvadze, Ketevan Chakhnashvili, Tamta Demurishvili, Paata Sabelashvili, Monique Foster, Liesl Hagan, Maia Butsashvili, Juliette Morgan, Francisco Averhoff

**Affiliations:** 1 Division of Viral Hepatitis, National Center for HIV/AIDS, Viral Hepatitis, STD, and TB Prevention, Centers for Disease Control and Prevention, 1600 Clifton Road NE, MS: G37, Atlanta, GA 30329 USA; 2 Infectious diseases, AIDS and Clinical Immunology Research Center, Tbilisi, Georgia; 3 Hubert Fellowship, Division of Viral Hepatitis, National Center for HIV/AIDS, Viral Hepatitis, STD, and TB Prevention, Centers for Disease Control and Prevention, Atlanta, GA USA; 4 Ministry of Corrections, Tbilisi, Georgia; 5 Global Disease Detection, Division of Global Health Protection, Centers for Disease Control and Prevention, Tbilisi, Georgia; 6 National Center for Disease Control and Public Health of Georgia, Ministry of Labour Health and Social Affairs (MoLHSA) of Georgia, Tbilisi, Georgia; 7 Ministry of Internal Affairs, Tbilisi, Georgia; 8 Treatment access activist, Tbilisi, Georgia; 9 Epidemic Intelligence Service, Centers for Disease Control and Prevention, Atlanta, GA USA; 10 Health Research Union / Clinic Neolab, Tbilisi, Georgia; 11 Division of Global Health Protection, Center for Global Health, Centers for Disease Control and Prevention, Atlanta, GA USA

**Keywords:** Chronic hepatitis C, HCV infection, Prisons, Global health security, Linkage to care, Incarcerated, Prisoner

## Abstract

**Background:**

The country of Georgia has a high burden of chronic hepatitis C virus (HCV) infection, and prisoners are disproportionately affected. During 2013, a novel program offering no cost screening and treatment of HCV infection for eligible prisoners was launched.

**Methods:**

The HCV treatment program implemented a voluntary opt-in anti-HCV testing policy to all prisoners. Anti-HCV positive persons received HCV RNA and genotype testing. Transient elastography was also performed on prisoners with positive HCV RNA results. Prisoners with chronic HCV infection who had ≥F2 Metavir stage for liver fibrosis and a prison sentence ≥ 6 months were eligible for interferon-based treatment, which was the standard treatment prior to 2015. We conducted an evaluation of the HCV treatment program among prisoners from the program’s inception in December 2013 through April 2015 by combining data from personal interviews with corrections staff, prisoner data in the corrections database, and HCV-specific laboratory information.

**Results:**

Of an estimated 30,000 prisoners who were incarcerated at some time during the evaluation period, an estimated 13,500 (45%) received anti-HCV screening, of whom 5175 (38%) tested positive. Of these, 3840 (74%) received HCV RNA testing, 2730 (71%) tested positive, and 880 (32%) met treatment eligibility. Of these, 585 (66%) enrolled; 405 (69%) completed treatment, and 202 (50%) achieved a sustained virologic response at least 12 weeks after treatment completion.

**Conclusions:**

HCV infection prevalence among Georgian prisoners was high. Despite challenges, we determined HCV treatment within Georgian Ministry of Correction facilities was feasible. Efforts to address HCV infection among prison population is one important component of HCV elimination in Georgia.

## Background

There are an estimated 71 million people infected with hepatitis C virus (HCV) and 399,000 associated deaths annually worldwide [1, 2]. Georgia is a lower-middle income country located in Eastern Europe, with a population of 3.7 million people and has one of the highest prevalence rates of HCV infection in the world [3]. In 2002, data from a serosurvey found 6.7% of the adult population in the capital city of Tbilisi had antibodies to HCV (anti-HCV) [3, 4]. A recent national serosurvey in 2015 estimated a 7.7% anti-HCV prevalence [5]. Estimates of anti-HCV prevalence among high-risk groups include: 57–92% among people who inject drugs (PWID), 17% among men who have sex with men, and 4–12% among health care workers [6]. Injection-drug use (IDU) is an important risk factor for HCV transmission in Georgia and the most common reason for incarceration [6]. Anti-HCV prevalence among prisoners in most countries is significantly higher than the prevalence in the general population [7–9].

Complaints of inadequate healthcare provided in Georgian prisons led to proceedings adjudicated in 2009 by the European Court of Human Rights, resulting in judgements against Georgia. The Court directed the Georgian government to provide prisoners with access to hepatitis C prevention and treatment and undertake systematic steps to ensure access to testing and treatment [10]. Immediately following elections in 2012, the new administration prioritized prison healthcare as a priority: The “18 months prison healthcare reform” was launched in 2013 and was successfully completed (according to an EU independent evaluation) in 2014. Introduction of the hepatitis C program in prisons was an important part of the prison healthcare reform; providing hepatitis C prevention counseling, testing and treatment services to inmates at no cost.

In this report, we evaluate the effectiveness of the hepatitis C treatment program in Georgian prisons. This evaluation provided an important opportunity to assess the program and through the lessons learned strengthen public health capacity. This will lead to improvements in the prevention and treatment of HCV in Georgia and globally, and thereby enhance global health security. It is anticipated that the challenges and successes identified in this evaluation would be used by public health policy makers to implement a successful prison treatment program which would contribute significantly toward Georgia’s national HCV elimination program, which began in April 2015 [11].

## Methods

Data were obtained from three sources: 1) Personal communication and interviews with Georgia Ministry of Corrections (MOC) officials; 2) A database maintained by the Georgian MOC that contains prisoner demographic information (age, sex, length of prison sentence, anti-HCV result, liver elastography score, and treatment received); and anti-HCV positive prisoners had additional blood samples sent to a laboratory, where a 3) database maintained by the Infectious Diseases, AIDS and Clinical Immunology Research Center (IDACIR) in Tbilisi that contains laboratory information from prisoners (HCV RNA result, HCV genotype, aspartate aminotransferase [AST], alanine aminotransferase [ALT], and platelet count). Georgian MOC officials merged data from these sources using prisoners’ names into one dataset for programmatic analysis, and was de-identified to ensure confidentiality.

### Description of the Georgia MOC HCV treatment program

The penitentiary system in Georgia consists of one female prison and ten male prisons and houses approximately 10,000 prisoners at any given time with a maximum capacity of 21,398. The majority of prisoners are male (97%), and 80% are aged 18–45 years.

The MOC launched a program for hepatitis C screening, care and treatment in Georgia’s prison system in December 2013. The MOC implemented a voluntary opt-in anti-HCV testing policy to all prisoners. Those who tested positive were offered confirmatory HCV RNA testing and, if positive, received non-invasive liver fibrosis staging with transient elastography (elastography). Liver elastography scores were recorded as categorical liver fibrosis scores that corresponded to Metavir stage; higher liver elastography scores indicate more liver fibrosis. Demographic information and liver fibrosis score were entered into a MOC database. Laboratory testing was only performed on persons with a liver elastography score corresponding to F2 or greater, and included: liver transaminases, platelet count, serial HCV RNA, and HCV genotype, which were entered into the IDACIR database.

Treatment eligibility criteria included: 1) Chronic HCV infection determined by detection of virus by PCR (HCV-RNA-positive) and HCV genotype test; 2) Transient elastography measurement ≥F2; and 3) Prison sentence long enough to complete the treatment, which was usually longer than 6 months. If a prisoner met these criteria, a committee composed of physicians from the MOC and the Ministry of Labour, Health, and Social Affairs, and representatives from community organizations reviewed each case, including medical and psychiatric records to identify any contraindications to interferon-based treatment regimens. After review, a determination was made as to whether the prisoner was eligible for the treatment program. The physicians on that committee determined the specific HCV treatment regimen to administer to each prisoner based on the American Association for the Study of Liver Diseases (AASLD) 2009 Practice Guidelines [12]. Treatment medications during the evaluation period included pegylated interferon and ribavirin for 24 or 48 weeks depending on the HCV genotype. The program had resources to provide treatment to 500 prisoners free of charge each year. Interferon-free regimens were not available in Georgia prior to April 2015.

### Statistical analysis

We described the HCV care cascade among prisoners by calculating the number of prisoners who: a) received anti-HCV testing; b) received confirmatory HCV-RNA and HCV genotype testing, and liver elastography score; c) were deemed eligible for treatment; d) enrolled in HCV treatment; e) began and completed their prescribed treatment course; and f) achieved a sustained virologic response (undetectable HCV RNA) at least 12 weeks post therapy (SVR12). The proportion achieved for each step was calculated using the preceding value as the denominator. We described the demographic characteristics, HCV genotype, and non-invasive liver fibrosis assessments for chronically infected prisoners who were treatment eligible. We calculated other non-invasive fibrosis assessments using the fibrosis-4 (FIB-4) score and AST to Platelet Ratio Index (APRI) for those who had laboratory data available. The FIB-4 score was calculated using the formula: (age [years] x AST [U/L]) / (platelets [10^9^/L] x square root ALT [U/L]) in which the age of the patient was the age at the time of the blood draw. FIB-4 scores < 1.45 have a negative predictive value of 90% for advanced fibrosis and scores > 3.25 have a 65% positive predictive value for F3/4 [13]. APRI was calculated using the formula: (AST [IU/L] / AST upper limit of normal [37 IU/L] / platelet count [10^9^/L]) × 100. The lower the APRI score (< 1.0) the greater the negative predictive value, and scores > 2.0 have a specificity of 91% for identifying cirrhosis [14]. Analyses were conducted using SAS Institute Inc. version 9.3 (Cary, NC, USA).

## Results

**Fig. 1:**
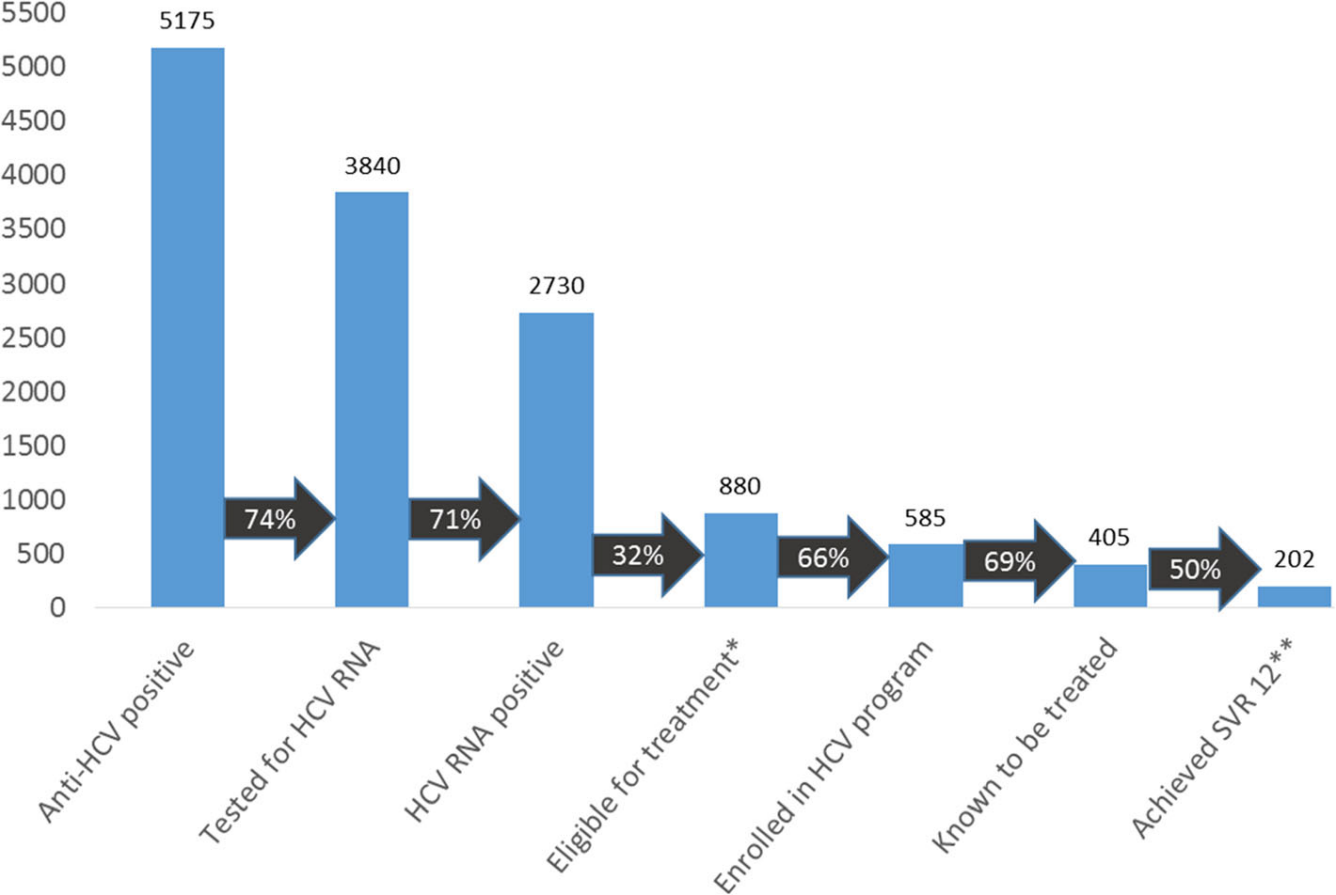
Chronic Hepatitis C Virus Infection Care Cascade Among Prisoners in Georgian Prisons, December 2013–April 2015. *Eligible for treatment required a transient elastrography result F2 or greater and a prison sentence long enough to complete treatment. **SVR = sustained virologic response, defined as not detectable HCV RNA at least 12 weeks after completion of therapy

This assessment included data from the program’s inception in December 2013 through April 2015. The total number of prisoners housed by MOC during the evaluation period was difficult to ascertain, but the MOC estimates 30,000 persons were in the prison system at some time during the evaluation period. Figure 1 illustrates the HCV care cascade. An estimated 13,500 (45%) prisoners received anti-HCV testing, and 5175 (38%) tested positive. Of those who tested positive, 3840 (74%) had confirmatory HCV RNA testing performed, and of those who had RNA testing, 2730 (71%) tested positive and were diagnosed with chronic HCV infection. Of 2730 prisoners diagnosed with chronic HCV infection, 880 (32%) met the eligibility criteria for treatment. Of these, 858 (98%) were male, 155 (18%) had elastography ≥ 12.5 consistent with Metavir F4, and most were infected with HCV genotype 3 (48%). Other characteristics are listed in the Table 1. FIB-4 and APRI identified 52 and 62 prisoners with advanced fibrosis or cirrhosis, respectively (Table 1). The strength of the agreement for liver elastography was moderate for FIB-4 (kappa = 0.568; 95% CI 0.456 to 0.679) and APRI (kappa = 0.545; 95% CI 0.437 to 0.653).

**Table 1:** Demographics, hepatitis C genotype, and non-invasive fibrosis assessment among Georgian prisoners with chronic hepatitis C infection, December 2013–April 2015

	Among prisoners receiving a full diagnostic evaluation	%
N	880	
Median age (years)	40 (Range: 18–71)	
Male (years)	40 (Range: 18–71)	
Female (years)	43 (Range: 25–54)	
Sex		
Male	858	97.5%
Female	22	2.5%
HCV Genotype		
1	200	22.7%
2	253	28.8%
3	420	47.7%
Mixed 1 & 2	5	0.6%
Mixed 1 & 3	1	0.1%
6	1	0.1%
Non-Invasive Fibrosis Staging		
6.5 kPa – < 8.0 kPa (Metavir F2)	406	46.1%
8.0 kPa – < 10 kPa (Metavir F2-F3)	192	21.8%
10 kPa – < 12.5 kPa (Metavir F3)	127	14.4%
12.5 kPa – < 14 kPa (Metavir F3-F4)	23	2.6%
≥ 14 kPa (Metavir F4)	132	15.0%
FIB-4		
< 1.45	573	65.1%
1.45–3.25	255	29.0%
> 3.25	52	5.9%
APRI		
< 1.0	651	74.0%
1.0–2.0	167	19.0%
> 2.0	62	7.0%

Of treatment eligible prisoners, 585 (66%) enrolled in treatment (Fig. 1). Of these, 405 (69%) had completed the full treatment course by the end of the evaluation period. Reasons for 180 prisoners with incomplete treatment data included: 125 (21%) were released from prison prior to treatment completion, 29 (5%) stopped treatment due to side effects or voluntary cessation, 4 (< 1%) stopped treatment due to lack of virologic response, and 22 (4%) were unknown.

Of 405 treated prisoners with HCV RNA results available, 365 (90%) achieved end of treatment response, and 202 (50%) achieved a SVR12.

## Discussion

Georgia’s prison population represents 0.3% of the total national population, and the anti-HCV prevalence was 38% in our program. Our findings support the feasibility of HCV treatment in Georgia’s penitentiary system. Specifically, the program screened one-third of prisoners for HCV within the first 2 years of its operation, enrolled 21% (585/2730) of those identified with chronic HCV infection in treatment, and achieved a sustained virologic response in at least 50% (202/405) of prisoners treated with interferon-based therapy. This program highlights the high demand for treatment among prisoners, as well as a strong commitment within the MOC and the Georgian government overall to reduce the burden of HCV infection within the prison system. With the introduction of newer, more effective, all-oral direct acting antiviral (DAA) regimens in Georgia starting in April 2015, the program’s effectiveness will likely increase and contribute to the government’s recent commitment to HCV elimination throughout the country [11].

The 38% anti-HCV prevalence reported in this evaluation is consistent with prevalence estimates reported by studies performed in prisons in the United States, which range from 17 to 41% [8, 9], and in Central Asia where prevalence has been documented at 38% [15]. A recent estimate of the global HCV prevalence among 10.2 million people incarcerated on any given day in 2014 was 15.1%, but authors noted geographic differences and HCV prevalence as high as 30% in Eastern Europe and central Asia [16]. Because of the high prevalence of HCV infection, correctional facilities are ideal locations to conduct screening and treatment programs because a large proportion of persons screened will test positive for chronic HCV infection [17]. High HCV infection prevalence in prisons is likely the result of a concentration of persons who inject drugs (PWID), as drug use is a major cause of incarceration in Georgia, and injection drug use is well recognized as a primary mode of HCV transmission [6, 18]. A meta-analysis estimated the incidence of HCV infection among incarcerated persons in 39 countries at 6.6 per 100,000 detainees with a history of IDU and 0.4 per 100,000 detainees without IDU [15]. In addition, a study in Scotland estimated HCV prevalence to be 49% among injector-inmates, and the HCV prevalence was 53% in those who had injected inside prison [19]. Further, IDU, as well as other risk factors, are prevalent in prisons and contribute to ongoing transmission within the prisons themselves [15]. HCV treatment programs similar to that pioneered by the Georgian MOC has the potential to reduce the burden of HCV infection within prisons, as well as contribute substantial public health impact by slowing the country’s overall HCV epidemic.

Early results from Georgia’s HCV prison program also demonstrate its ability to support successful completion of HCV treatment, as more than 70% of prisoners who initiated treatment completed their treatment course. Of the prisoners unable to complete their prescribed regimen, the majority discontinued due to tolerability issues at a rate lower or comparable to non-institutionalized populations [20, 21]. Drop-out rates are likely to decrease with the introduction of newer, all-oral interferon-free DAA regimens. In addition, some prisoners decided to defer interferon-based treatment and wait until the interferon-free regimens were available. We hypothesize that integrating these new regimens would lead to an even higher impact on reducing HCV infection prevalence in Georgia’s prison system.

Despite these early successes, there are areas for improvement in Georgia’s current HCV prison program. First, due to the opt-in structure of the screening component, less than half of prisoners received anti-HCV testing during the evaluation period. To overcome this challenge, the MOC could adopt an opt-out structure. Second, there were 1335 (26%) anti-HCV positive prisoners who did not receive confirmatory HCV-RNA PCR testing, which may have resulted in an underestimated burden of chronic HCV infection in Georgian prisons. A second blood draw is required to perform HCV RNA testing which may have been a contributory factor. Reflex HCV RNA testing could overcome this barrier. Third, more than half of prisoners with chronic infection did not receive full diagnostic evaluation including non-invasive fibrosis staging. This gap may have led to under treatment of eligible prisoners with chronic HCV infection and could be overcome by performing a comprehensive non-invasive liver fibrosis staging work up in all chronically infected prisoners. In the recently released World Health Organization guidance for HCV, easy to implement non-invasive liver fibrosis scores including FIB-4 and APRI are recommended for liver fibrosis staging [22]. FIB-4 and APRI have been shown to have high sensitivities for identifying persons without cirrhosis [23]. Our data showed moderate agreement for identifying prisoners with advanced fibrosis or cirrhosis using FIB-4/APRI compared with liver elastography. Utilization of other non-invasive liver fibrosis scoring tools could be considered as a screening tool for advanced liver fibrosis in future programs. Fourth, 79% of Georgian prisoners with chronic HCV infection did not enroll in treatment, due to strict eligibility criteria. This barrier could be mitigated by adjusting eligibility criteria to reflect the shorter treatment duration (≤12 weeks) possible with interferon-free regimens recently introduced in Georgia, which will allow prisoners with shorter prison sentences to participate. Further investigation is needed to evaluate interventions to mitigate these gaps in the HCV care cascade [11].

Prevention and education are also necessary components for a successful hepatitis C control program in Georgian prisons. For example, an HCV treatment program in Australian prisons reported that 5 of 57 successfully treated prisoners became re-infected [24], indicating that comprehensive prevention strategies including harm reduction and addiction services are crucial for hepatitis C burden reduction and eventual elimination. The Georgian HCV prison program provides risk reduction education to prisoners, including counseling and methadone therapy if needed, but the effectiveness of these programs was not assessed in this evaluation.

The data from this evaluation show that genotype 3 was the predominant HCV genotype among Georgian prisoners during the evaluation period, consistent with a recent respondent-driven-sampling study of PWID that found that 67% were infected with genotype 3 [18]. A national HCV serosurvey conducted in 2015 found higher prevalence of genotype 1 infection (41% of those with a positive HCV RNA test) compared to 35% with genotype 3 in the general population [5]. These studies indicate that there could be systematic differences in the dynamics of the HCV infection epidemic in the prison system compared to the general population, including risk factors for transmission. The HCV genotype distribution among prisoners impacts choice of treatment regimen and is important to consider when estimating associated costs to payers. Specifically, under the treatment regimens used in Georgian prisons during this evaluation period, treatment duration for genotype 3 infections was half that required for genotype 1 (24 vs. 48 weeks, respectively) and therefore less expensive. The distribution of HCV genotypes among prisoners may have cost considerations in the context of Georgia’s HCV elimination strategy.

Our evaluation had several limitations. First, data were abstracted from multiple sources, and some important variables, including prisoners’ birth date, individual risk factor data, and treatment committee decisions for those prisoners who were not offered treatment were missing. A single database with a comprehensive set of HCV-related variables would improve monitoring strategies in the future. Second, not all prisoners infected with HCV received a full diagnostic evaluation including non-invasive liver fibrosis staging, potentially resulting in under treatment of eligible prisoners. Third, treatment data were not available for all prisoners completing treatment, thus limiting our evaluation of treatment success. Fourth, costs for the program were not assessed, and could inform future policy. Finally, since this was a retrospective analysis, we were not able to perform quality assurance and quality control on the data collected.

## Conclusions

In conclusion, this evaluation demonstrates that a HCV treatment program within the Georgian prison system is feasible, as the majority of prisoners enrolled in treatment in the first 2 years of this program’s operation were able to complete their prescribed treatment course. This evaluation also provided an important opportunity to strengthen the public health capacity of Georgia, and thereby enhance global health security. There are several opportunities to enhance the success of the HCV treatment program in the Georgian prison system in the future. Specifically, an opt-out anti-HCV screening structure would further increase identification of infection, and use of newly introduced interferon-free regimens could improve treatment enrollment, adherence, efficacy, and completion. Offering linkage to community-based care to prisoners with short sentences could improve enrollment and completion rates as well. In addition, improved health information data systems would allow for optimal evaluation of future programs. Because most prisoners are eventually released and reintegrated into the community, HCV treatment and prevention in prisons can reduce the HCV infection burden in the general population, contributing to Georgia’s overall goal of HCV elimination and serving as a model for other countries pursuing similar targets.

## Abbreviations

ALT: Alanine aminotransferase
Anti-HCV: Antibody to hepatitis C virus
APRI: Aspartate aminotransferase to platelet ratio index
AST: Aspartate aminotransferase
DAA: Direct acting antiviral
FIB-4: Fibrosis-4 score
Fx: Liver fibrosis stage
HCV Genotype: Hepatitis C virus genotype
HCV RNA: Hepatitis C virus ribonucleic acid
HCV: Hepatitis C virus
IDACIR: Infectious Diseases, AIDS and Clinical Immunology Research Center
IDU: Injection drug use
IU/L: International units per liter
MOC: Ministry of corrections
PCR: Polymerase chain reaction
PWID: People who inject drugs
SVR12: Sustained virologic response 12 weeks after completion of treatment
